# Immunohistochemical detection of the transcription factor osterix in canine and feline osteosarcoma

**DOI:** 10.1177/03009858261457962

**Published:** 2026-06-30

**Authors:** Katherine Ings, Stephanie Plog, Mark Jackson, Francesco Marchesi, Angie Rupp

**Affiliations:** 1 Division of Pathology, Public Health and Disease Investigation, School of Biodiversity, One Health and Veterinary Medicine, College of Medical, Veterinary and Life Sciences, University of Glasgow, Glasgow, UK; 2 IDEXX Laboratories, Wetherby, UK; 3 School of Cancer Sciences, College of Medical, Veterinary and Life Sciences, University of Glasgow, Glasgow, UK

**Keywords:** bone tumor, cat, dog, immunohistochemistry, osteoblast, osteosarcoma, osterix, SP7

## Abstract

Osteosarcoma (OSA) is the most common primary bone tumor in dogs and cats, and accurate diagnosis of OSA has therapeutic and prognostic implications. Immunohistochemistry targeting osterix (Osx), a transcription factor expressed in osteoblasts, was applied to 80 canine samples (40 OSAs and 40 controls (8 chondrosarcomas, 8 fibrosarcomas, 8 hemangiosarcomas, 8 histiocytic sarcomas, and 8 malignant melanomas)) and 40 feline samples (20 OSAs and 20 controls (5 fibrosarcomas, 5 hemangiosarcomas, 5 chondrosarcomas, 3 malignant melanomas, and 2 histiocytic sarcomas)), diagnosed histologically. All 120 samples were assessed blinded using a previously established semiquantitative scoring and classification system. In dogs, 37/40 OSAs (true positives, sensitivity 92.5%) and 2/40 controls (false positives, specificity 95.0%) were classified as OSA. In cats, 14/20 OSAs (true positives, sensitivity 70.0%) and 5/20 controls (false positives, specificity 75.0%) were classified as OSA. Tumors most often “misclassified” as OSAs were chondrosarcomas in both dogs (2/8) and cats (4/5). In 4 cases for which paired decalcified and nondecalcified samples of the same OSA were available, decalcification of OSAs did not affect classification. Quiescent canine, feline, and murine osteoblasts and canine reactive, metaplastic, and non-OSA neoplastic osseous conditions were also tested and demonstrated Osx immunoreactivity; avian and piscine osteoblasts did not. In conclusion, Osx is a highly sensitive and specific marker of canine OSA and a moderately sensitive and specific marker of feline OSA. The diagnostic algorithm applied in this study is a useful adjunctive tool in the diagnosis of canine OSA.

Osteosarcoma (OSA) is a malignant tumor of osteoblasts that occurs in various species and is most often diagnosed in dogs, followed by cats.^37^ OSAs are notoriously pleomorphic tumors, and histological confirmation of osteoid produced by malignant osteoblasts is essential for a definitive diagnosis of OSA.^37^ However, osteoid can be scant or unevenly distributed within the tumor, and small biopsy samples may not contain osteoid. Additionally, samples of osseous and nonosseous lesions, such as malignant melanomas, may include reactive or metaplastic bone formation, respectively, further complicating the diagnosis of OSA.

Immunohistochemical approaches have been evaluated to facilitate the diagnosis of OSA. One such previous investigation indicated a high sensitivity (83%) of an anti-osteocalcin antibody in identifying canine OSA; however, in most cases, osteoid failed to demonstrate immunoreactivity, and immunohistochemistry (IHC) for osteocalcin did not allow distinction between OSA and chondrosarcoma.^42^ On the contrary, IHC for osteonectin labeled all of the OSAs, as well as the chondrosarcomas, fibrosarcomas, histiocytic sarcomas, and hemangiosarcomas tested, indicating a complete lack of specificity.^42^ A different study investigated the use of antibodies against alkaline phosphatase, osteonectin, osteopontin and RUNX2, and reported RUNX2 (specificity: 78%; sensitivity: 87%) to be the most specific and alkaline phosphatase the most sensitive at 100% in identifying canine OSA; however, the specificity of alkaline phosphatase was only 30%.^4^ Finally, anti-special AT-rich sequence-binding protein 2 antibody has been used as part of an immunohistochemical staining panel to identify canine OSA, for which the sensitivity was 86.0% and the specificity was 98.8%.^33^

In contrast to investigations conducted on canine tissues, IHC studies featuring feline OSAs are rare, although single case reports, including one indicating osteocalcin immunoreactivity, have been published.^7^ Thus, there is currently no single sensitive and specific IHC marker for OSA in dogs and very little evidence for suitable markers in cats.

Osterix (Osx), also known as SP7, is a transcription factor involved in osteoblast differentiation and bone formation.^6,30^ Both RUNX2 and Osx are required for osteoblast differentiation in mammals. RUNX2 is expressed early during osteochondroprogenitor differentiation, and Osx is expressed later, during osteoblast maturation, when Osx induces RUNX2-expressing precursor osteoblasts to differentiate into mature, functional osteoblasts.^2,34^ *Osx*-null mice have a normal cartilaginous skeleton but defective bone formation, and conditional knockout of Osx expression in cartilage confirmed a developmental role of Osx in coupling chondrogenesis and osteogenesis.^6,22,30^ In addition, conditional deletion of the *Osx* gene in chondrocytes in the early postnatal period disrupts secondary ossification of the tibial epiphysis,^22,45^ and Osx has also been shown to be important in postnatal bone growth and homeostasis of the adult skeleton.^3^ In summary, Osx plays a critical role in osteoblast differentiation in bone development and maintenance.

The expression of Osx has been investigated by IHC in a range of human bone tumors including OSAs, where it was found to be of minimal diagnostic utility due to low frequency of labeling, although immunoreactivity was restricted to OSAs.^20^ Immunohistochemical detection of Osx has been reported in extraskeletal OSAs in a rat, a mouse, and a rabbit and in a vertebral giant cell tumor of bone in a cat.^5,21,28,43^

This study hypothesized that immunohistochemically detectable expression of the transcription factor Osx would allow identification of canine and feline OSAs and distinguish them from salient differentials. To investigate this, anti-Osx antibody was applied to canine and feline OSAs, fibrosarcomas, chondrosarcomas, hemangiosarcomas, malignant melanomas, and histiocytic sarcomas. In addition, anti-Osx antibody was applied to a small selection of non-neoplastic and neoplastic osseous samples from various veterinary species.

## Materials and Methods

This study received ethical approval from the School of Biodiversity, One Health and Veterinary Medicine, University of Glasgow, Research Ethics Committee (EA61/23).

### Selection of Cases

The database of Veterinary Diagnostic Services (University of Glasgow, UK) was searched for submissions between 1999 and 2021 diagnosed as canine or feline OSA as well as salient control (CTRL) processes: chondrosarcoma, fibrosarcoma, hemangiosarcoma, malignant melanoma, and histiocytic sarcoma. The search progressed in reverse chronological order until the required case/group numbers were reached. Case/group numbers for canine tissues were set prior to selection of cases following consultation with a statistician and adjusted for feline tissues to reflect lower numbers of available OSAs. For all cases, all available slides were reviewed, and the most representative slide was chosen for consensus diagnosis, subtyping, and IHC. Study inclusion required a consensus diagnosis by 2 board-certified pathologists. If a consensus could not be reached, cases were reviewed by a third board-certified pathologist for a majority consensus. If a majority consensus was not reached, cases were omitted and the database was revisited, starting from where the previous search had stopped and continuing in reverse chronological order. One suitable CTRL case for the canine and feline cohort each was added as they came through the diagnostic service in 2022 and 2024 and before the final selection of cases underwent IHC.

In general, OSAs were selected based on the unequivocal presence of a malignant spindle cell population producing osteoid and subtyped based on established histological criteria into osteoblastic (productive or poorly productive), chondroblastic, fibroblastic, telangiectatic, and giant cell-rich OSAs.^36,38^ Where available, paired decalcified and nondecalcified samples of the same OSA (*n* = 4) were included. The histological inclusion criteria for CTRLs were as previously described.^14,18,27,39,41^ Histological investigations were, if required and/or possible, supplemented by IHC. For chondrosarcoma, samples comprised a malignant mesenchymal population producing chondroid matrix and lacking osteoid production. For fibrosarcoma, samples comprised a malignant spindle cell population producing collagen and lacking production of osteoid (and chondroid) matrix, commonly arranged in interwoven streams, occasionally also in herringbone patterns. For hemangiosarcoma, samples comprised a (CD31-positive) malignant spindle cell population lining irregularly shaped and sized blood-filled vascular spaces commonly supported by thin collagenous trabeculae and/or combined with more solid tumor cell aggregates. For malignant melanoma, samples comprised a (PNL2- and/or melanA-positive) malignant polygonal to spindeloid cell population containing variable amounts of intracytoplasmic melanin granules, with some samples representing poorly or amelanotic variants. Finally, for histiocytic sarcoma, samples comprised a commonly highly pleomorphic, polygonal to spindeloid, malignant neoplastic population of large (IBA1- and/or CD18-positive) mononuclear cells with multinucleated giant cells and bizarre mitotic figures, forming sheets and variably infiltrated by lymphocytes and neutrophils. Feline histiocytic sarcomas were typically slightly less pleomorphic.

Several additional samples comprising both neoplastic and non-neoplastic osseous samples from various species, often obtained from necropsy cases with varying postmortem intervals, were also selected, again based on a majority consensus diagnosis.

### Study Samples

The origin and distribution of the study’s OSAs are summarized in Table 1. The canine study population comprised 40 decalcified OSA samples (27 productive osteoblastic, 3 poorly productive osteoblastic, 6 chondroblastic, 3 fibroblastic, and 1 telangiectatic), of which 37 represented biopsy specimens and 3 represented necropsy specimens, and 40 CTRL samples (8 chondrosarcomas, 8 fibrosarcomas, 8 hemangiosarcomas, 8 histiocytic sarcomas, and 8 (bleached) malignant melanomas), all of which represented biopsy specimens. The feline study population comprised 20 decalcified OSA samples (7 productive osteoblastic, 3 poorly productive osteoblastic, 3 chondroblastic, 5 giant cell-rich, and 2 telangiectatic), of which 19 represented biopsy specimens and 1 represented a necropsy specimen, and 20 CTRL samples (5 fibrosarcomas, 5 hemangiosarcomas, 5 chondrosarcomas, 3 (bleached) malignant melanomas, and 2 histiocytic sarcomas), of which 19 represented biopsy specimens and 1 represented a necropsy specimen.

**Table 1.** Osteosarcoma study samples.

Species	Axial	Appendicular	Extraskeletal	Total
Canine	9	26	5	40
	6 skull 2 vertebra 1 chest^a^	9 (7 proximal, 1 distal) humerus 6 (5 distal) femur 5 distal radius 3 (1 distal) tibia 1 forelimb^a^ 1 distal ulna 1 metatarsus	1 jejunum 1 ileum 2 liver 1 subcutis	
Feline	7	11	2	20
	5 skull 1 chest^a^ 1 pelvis	3 (2 distal) scapula 2 distal femur 2 (1 proximal and 1 distal) tibia 1 forelimb^a^ 1 proximal humerus 1 carpus^a^ 1 hindlimb^a^	1 subcutis (interscapular) 1 subcutis (overlying scapula)	

a Exact bone(s) affected unclear.

### Additional Samples

To compare the effect of decalcification and bleaching on Osx immunoreactivity, 4 non-decalcified OSA biopsy samples (3 canine, 1 feline) and 11 unbleached malignant melanoma samples (8 canine, 3 feline) paired to the above study sample population were also scored as described below.

Finally, 1 example of each of the following entities was included in the assessment: canine meningioma with osseous metaplasia; canine calcinosis circumscripta with osseous metaplasia; canine pachymeningitis ossificans; canine and feline periosteal bone formation; canine Legg-Calvé-Perthes syndrome; feline osseous metaplasia; canine osteoma; canine multilobular tumor of bone; bovine ossifying fibroma; immature mouse (embryo, ~embryonic day 15) and kitten (1-week-old) bone; and healthy mature rat, mouse, fish (zebrafish [*Danio rerio*]), and avian (parrotlet [species unspecified] and chicken [*Gallus gallus domesticus*]) bone.

### Histology and Immunohistochemistry

All bony and substantially mineralized tissues in both the study sample and additional sample groups (except for the 4 OSA samples not requiring decalcification) were decalcified, following fixation in 10% neutral-buffered formalin and prior to histological processing. For this, tissues were placed in 90% formic acid (VWR, Avantor, UK; 95494.320) mixed with 20% sodium citrate (Fisher Chemical, Fisher Scientific, UK; S/3320/53) at a ratio of 35:65 and assessed daily for a maximum of 14 days until sufficiently demineralized for standard processing.

Following standard processing, blocks were sectioned at a thickness of 3 µm, and sections were stained with hematoxylin and eosin.

Optimization of the IHC for Osx was carried out on a non-study, decalcified dog tissue comprising a combination of normal bone and OSA (positive control), and soft tissues (collagen, adipose tissue, vessels), small aggregates of inflammatory cells, epidermis, and adnexal structures (negative controls). In this sample, no labeling was observed in any of the negative control tissues.

For IHC, 4-µm thick sections of formalin-fixed, paraffin-embedded tissue were mounted onto Slidemate Plus Adhesion Microscope Slides (Epredia, Fisher Scientific, UK), deparaffinized, and rehydrated in a graded alcohol series. Malignant melanomas were also bleached with 3% H_2_O_2_ (VWR, Avantor, UK; 23619.297). Antigen-retrieval was performed using heat-induced epitope retrieval, and sections were treated at full pressure with the Access Retrieval Unit MenaPath (model MP-2002-CE, Menarini, UK) in EDTA buffer (pH 9) for 90 seconds at 125°C, then rinsed in Tris Tween buffer (pH 7.5; Tween 20 for synthesis, Sigma Aldrich, UK; 8.22184). Endogenous peroxidase was quenched by incubation of sections for 5 minutes with 3% H_2_O_2_ in phosphate-buffered saline. After washing, sections were incubated for 30 minutes at room temperature with the primary rabbit monoclonal anti-Osx antibody (Abcam, UK; ab209484; 1:1000). For primary antibody detection, sections were incubated with EnVision+ System HRP-labeled polymer anti-rabbit secondary antibody (Dako, UK; K400311-2) for 30 minutes at room temperature. This was followed by two 5-minute incubations with 3,3’-diaminobenzidinesubstrate-chromogen (EnVision+ System; Dako, Agilent Technologies, Inc, UK). Following rinsing of sections with distilled water for 5 minutes, tissues were counterstained using Gill’s hematoxylin and coverslipped. Samples were run in the largest possible batches, resulting in a total of 7 runs, each of which included a (technical) negative control (omission of the primary antibody and incubation with diluent only).

Slides were scanned using Motic EasyScan Infinity 60 (Motic, Europe) at 40× magnification (lens specification: 20× 0.75 Plan Apochromat).

### IHC Scoring, Sample Classification, and Statistical Analyses

The malignant neoplastic population in the canine and feline OSAs (including the nondecalcified specimens available for a small number of masses) and CTRLs were annotated by an individual not involved in scoring and then scored by 2 blinded board-certified pathologists, in duplicate (Scorers 1 and 2) on scanned sections and once on glass slides (Scorer 2).

Nuclear IHC labeling of target neoplastic populations was quantified using a semiquantitative scale as previously described.^4^ Briefly, the assessment comprised 5 scores: 0—no nuclear labeling, 1—labeling of 1% to 15% of nuclei, 2—labeling of 16% to 40% of nuclei; 3—labeling of 41% to 75% of nuclei, and 4—labeling of 76% to 100% of nuclei. A score <2 was considered negative (non-OSA), a score ≥2 was considered positive (OSA).^4^ In cases where the positive/negative classification was at odds between the 2 scorers, the annotated slide was reviewed by a third blinded, board-certified pathologist (Scorer 3), and a majority consensus was reached.

The additional neoplastic and non-neoplastic osseous tissues from various additional species were assessed according to the extent and intensity of labeling in the cells of interest.

Sensitivity was defined as the proportion of OSAs (consensus diagnosis on hematoxylin and eosin) classified as OSA following IHC for Osx. Specificity was defined as the proportion of non-OSAs (consensus diagnosis on hematoxylin and eosin) classified as non-OSA following IHC for Osx. The proportion of correctly classified samples (accuracy) was determined and subjected to a one-sided binomial test against a no information rate of 0.5. Confidence intervals (CIs) (95%) were computed for test parameters using the Clopper-Pearson method. Correlations between scores were determined using Cohen’s weighted kappa with 95% CI.

The data analyzed in this study are available in Supplemental Table S1.

## Results

### Osx IHC in Canine and Feline OSAs and CTRLs

The anti-Osx antibody labeled the nuclei of the malignant spindle cell population producing osteoid in OSAs (Figs. 1b (dog) and 2b (cat)). In more strongly labeling specimens with a large proportion of the malignant spindle cell population producing osteoid demonstrating nuclear labeling, concurrent weaker cytoplasmic labeling was commonly observed (Fig. 1b).

**Figure 1.**
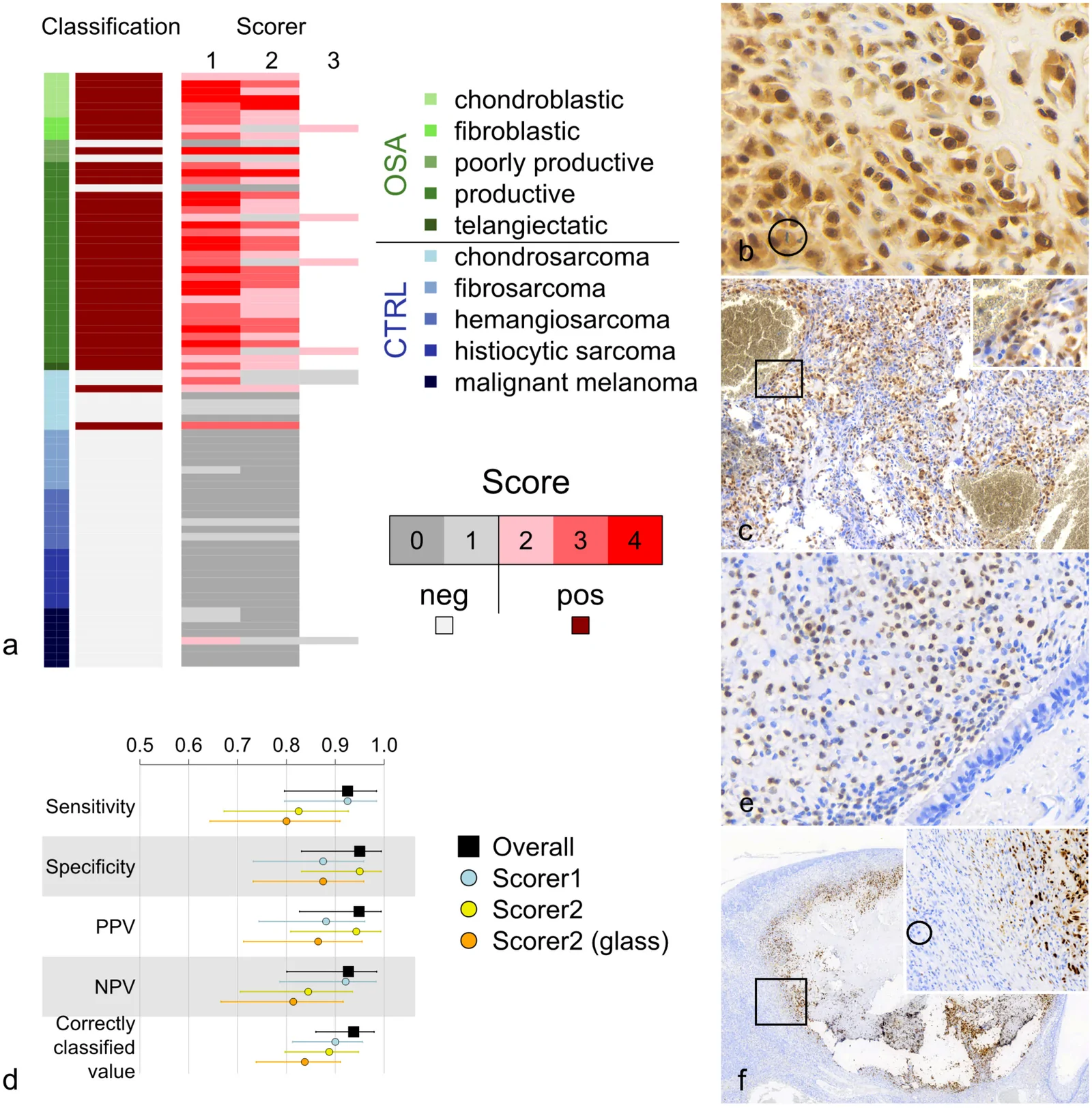
Immunohistochemistry (IHC) for osterix (Osx) in dogs. (a) Scoring and classification of canine osteosarcomas (OSA; *n* = 40) and control tumors (CTRL; *n* = 40). Following IHC for Osx, 37 OSAs and 2 CTRLs were classified as OSA (score ≥ 2), and 3 OSAs and 38 CTRLs were classified as non-OSA (score <2). (b) Canine productive osteoblastic OSA. Strong nuclear and weaker cytoplasmic immunoreactivity is observed in neoplastic osteoblasts. (Circle highlights a mitotic figure). (c) Canine osteosarcoma of telangiectatic subtype. Strong nuclear immunoreactivity is observed in a high proportion of neoplastic cells including those delineating vessel-like structures (box and corresponding inset). (d) Samples were classified according to combined (Scorers 1-3, overall) and individual scores, and classification performance parameters were computed with Clopper-Pearson 95% confidence intervals. In addition to digital scans, classification from glass slide scores was performed by Scorer 2. PPV, positive predictive value; NPV, negative predictive value. (e) Canine nasal chondrosarcoma. Strong nuclear immunoreactivity is observed in a substantial proportion of neoplastic chondroblasts. (f) Canine (bleached) oral malignant melanoma. The neoplastic population is not immunoreactive with anti-Osx antibody; however, strong nuclear immunoreactivity is observed in a region of osseous metaplasia (box and corresponding inset). (Circle highlights a mitotic figure in the melanocytic neoplastic population).

**Figure 2.**
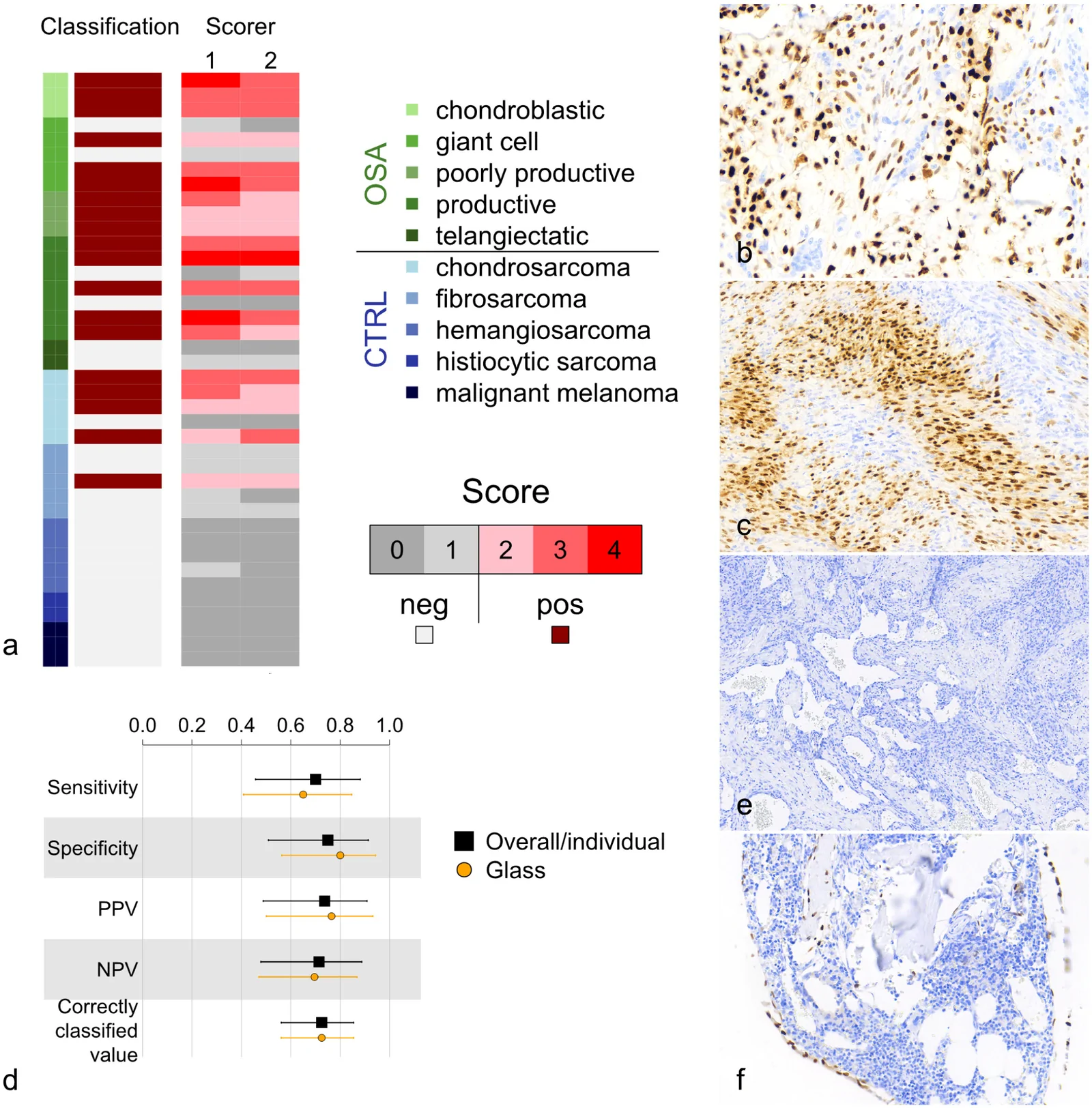
Immunohistochemistry (IHC) for osterix (Osx) in cats. (a) Scoring and classification of feline osteosarcomas (OSA; *n* = 20) and control tumors (CTRL; *n* = 20). Following IHC for Osx, 14 OSAs and 5 CTRLs were classified as OSA (score ≥2), and 6 OSAs and 15 CTRLs were classified as non-OSA (score <2). (b) Feline OSA of giant cell-rich subtype. Strong nuclear immunoreactivity is observed in the neoplastic osteoblasts but not in the multinucleated cells. (c) Feline subcutaneous fibrosarcoma. Strong nuclear immunoreactivity is observed in a substantial proportion of neoplastic fibroblasts. (d) Sample classification was identical between scorers, so single overall/individual classification performance values were computed with Clopper-Pearson 95% confidence intervals. In addition to digital scans, classification from glass slide scores was performed by Scorer 2. PPV, positive predictive value; NPV, negative predictive value. (e) Feline subcutaneous hemangiosarcoma. No immunoreactivity is observed. (f) Mature feline bone. Strong nuclear immunoreactivity is observed in osteoblasts and rarely in osteocytes.

Immunohistochemical scoring and classification of all 80 scanned canine samples (40 OSAs, 40 CTRLs) resulted in 39 samples (37 OSAs and 2 CTRLs) being classified as OSA and 41 samples (3 OSAs and 38 CTRLs) being classified as non-OSA (Fig. 1a, b, c, e, f). This yielded an overall sensitivity of 92.5% (95% CI = 79.6% to 98.4%), a specificity of 95.0% (95% CI = 83.1% to 99.4%), a positive predictive value of 94.9% (95% CI = 82.7% to 99.4%), a negative predictive value of 92.7% (95% CI = 80.1% to 98.5%), and a correctly classified value of 93.8% (95% CI = 86.0% to 97.9%, *P* < .0001) for Osx as an immunomarker in assessing canine OSAs and salient differentials (Fig. 1d). The 3 canine OSAs not classified as OSA were poorly productive (*n* = 2) and productive (*n* = 1) subtypes, whilst the 2 CTRLs classified as OSA were both nasal chondrosarcomas (Fig. 1a, e).

In the canine cohort, productive and chondroblastic OSAs were typically allocated higher scores when compared to other OSA subtypes, whilst poorly productive OSAs exhibited the greatest score variability and were the subtype most often classified as non-OSA (score <2). Of the CTRLs, chondrosarcomas exhibited the highest scores and were most often classified as OSA (score ≥2; Fig. 1a, e).

Immunohistochemical scoring and classification of all 40 scanned feline samples (20 OSAs, 20 CTRLs) resulted in 19 samples (14 OSAs and 5 CTRLs) classified as OSA and 21 samples (6 OSAs and 15 CTRLs) classified as non-OSA (Fig. 2a, b, c, e). This gave an overall sensitivity of 70.0% (95% CI = 45.7% to 88.1%), a specificity of 75% (95% CI = 50.9% to 91.3%), a positive predictive value of 73.7% (95% CI = 48.8% to 90.9%), a negative predictive value of 71.4% (95% CI = 47.8% to 88.7%), and a correctly classified value of 72.5% (95% CI = 56.1% to 85.4%, *P* = .0032) for Osx as an immunomarker when assessing feline OSAs and salient differentials (Fig. 2d). The 6 feline OSAs not classified as OSA were productive (*n* = 2), giant cell-rich (*n* = 2), and telangiectatic (*n* = 2) subtypes, whilst the 5 feline CTRLs classified as OSA were chondrosarcomas (*n* = 4; locations: nose, hindlimb, axilla, and subcutis of flank) and a fibrosarcoma (*n* = 1; location: subcutis of flank; Fig. 2a, c).

In the feline cohort, productive and chondroblastic OSAs typically scored higher than other OSA subtypes, but productive OSAs also demonstrated the greatest variability of scores. Multinucleated cells in OSAs of the giant cell-rich subtype lacked labeling (Fig. 2b), and both telangiectatic OSAs under investigation were classified as non-OSA (score <2). Of the CTRLs, chondrosarcomas exhibited the highest scores and were most often classified as OSA (score ≥2; Fig. 2a).

### Scorer Agreement and Scoring System

Correlations (Cohen’s weighted kappa) between score values (Scorer 1 vs Scorer 2) were 0.85 (0.79-0.90) and 0.92 (0.88-0.96) for canine (*n* = 80) and feline (*n* = 40) samples, respectively. Interscorer classification agreement (Scorer 1 vs Scorer 2) values were 91.3% and 100% for the canine (Fig. 1a) and feline (Fig. 2a) tissues, respectively, translating to broadly similar (canine) or identical (feline) scorer classification performance parameters (sensitivity, specificity, positive and negative predictive values, and correctly classified value; Figs. 1d and 2d).

Assessment of classification receiver operator characteristics revealed markedly similar areas under the curve for the individual scorers in both canine (Scorer 1: 0.941; Scorer 2: 0.944) and feline (Scorer 1: 0.787; Scorer 2: 0.783) tissues. In addition, the use of cut-off ≥2 was optimal for the classification of both canine and feline OSAs using this scoring system.

### Comparison of Glass vs Scanned IHC Slides

The weighted score correlation for glass vs scanned IHC slides (*n* = 120) assessed by the same observer (Scorer 2) was 0.92 (0.89 to 0.95) and was almost identical for canine (*n* = 80; 0.92, 0.89 to 0.95) and feline (*n* = 40; 0.92, 0.87 to 0.96) tissues. The classification agreement between samples assessed on glass vs scanned slides for Scorer 2 was 92.5% for dogs, 95.0% for cats, and 93.3% overall, which correspondingly translated to similar classification performance parameters (sensitivity, specificity, positive and negative predictive values, and correctly classified value) in both dogs and cats (Figs. 1d and 2d).

### Decalcification and Bleaching

Paired decalcified and non-decalcified samples from OSAs (3 canine, 1 feline) were assessed for Osx immunoreactivity. Scores given to non-decalcified samples were higher than or equal to those given to decalcified samples; however, this did not result in a change in classification (OSA vs non-OSA) regardless of scorer or sample type (glass vs scanned slides). Furthermore, there were no differences in scoring (or classification) of 10 malignant melanoma samples (7 canine, 3 feline) examined on glass or scanned slides for Osx immunoreactivity before and after bleaching; 1 canine sample could not reliably be assessed due to the extent of pigmentation.

### Observations in Additional Samples

IHC for Osx also resulted in strong nuclear labeling, which was often combined with moderate cytoplasmic labeling of quiescent mature feline (Fig. 2f), canine (Fig. 3a), and murine (mouse and rat; Fig. 3b) osteoblasts, and osteoblasts in the context of (canine and feline) reactive and woven bone formation (Fig. 3c, d). Furthermore, canine and bovine, and to a lesser extent feline cells in the context of osseous and chondroid metaplasia exhibited moderate to strong nuclear Osx immunoreactivity and weaker cytoplasmic immunoreactivity (Figs. 1f and 3e). In canine osteoma, the neoplastic population exhibited strong nuclear Osx immunoreactivity, rarely combined with weak cytoplasmic immunoreactivity (Fig. 3f), whilst in canine multilobular tumor of bone, plump neoplastic spindle cells directly lining the osseous and cartilaginous islands and, to a lesser extent, cells within islands with chondrocyte/chondroblast morphology demonstrated moderate to strong nuclear labeling, occasionally combined with weaker cytoplasmic labeling following IHC for Osx (Fig. 3g).

**Figure 3.**
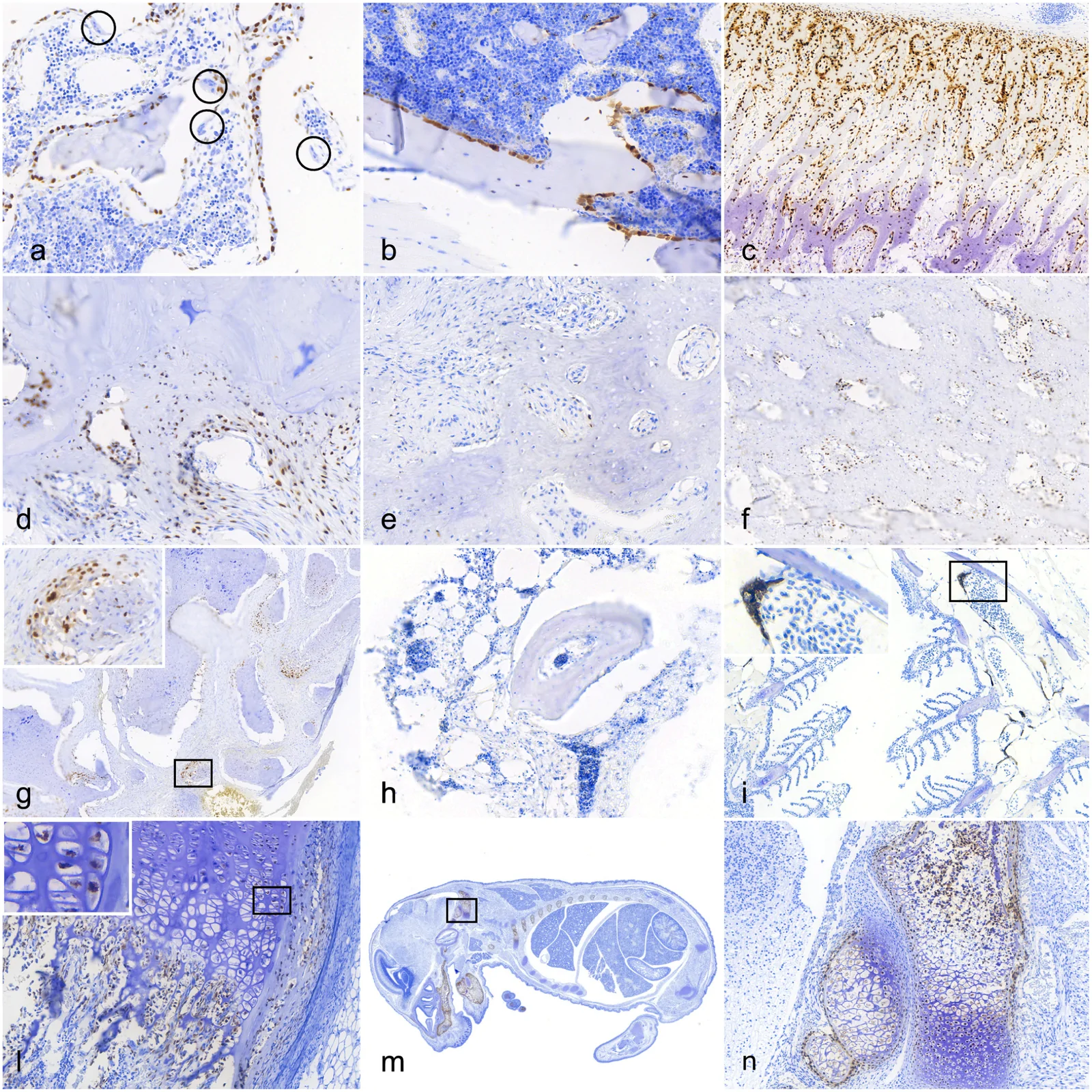
Immunohistochemistry for osterix (Osx) in various species and conditions. (a) Mature canine bone. Strong nuclear immunoreactivity is observed in osteoblasts; only small numbers of osteocytes exhibit nuclear immunoreactivity, and osteoclasts (circled) lack immunoreactivity. (b) Mature mouse bone. Strong nuclear and weaker cytoplasmic immunoreactivity is observed in osteoblasts, and rarely in osteocytes. (c) Feline periosteal bone formation. Strong nuclear immunoreactivity is observed. (d) Canine bone remodeling. Strong nuclear immunoreactivity is observed in osteoblasts and osteocytes associated with woven bone. (e) Feline metaplastic bone formation. Occasional strong nuclear immunoreactivity is observed delineating regions of metaplastic bone formation. (f) Canine osteoma. Strong nuclear immunoreactivity is observed in osteoblasts and less often in osteocytes. (g) Canine multilobular tumor of bone. Strong nuclear immunoreactivity is observed in a small proportion of neoplastic cells (box and corresponding inset). (h) Mature chicken bone. No immunoreactivity is observed. (i) Mature zebrafish. No immunoreactivity is observed in bones and cartilage associated with and surrounding the gills. (The pigment represents melanin, box and corresponding inset). (l) Immature feline femur. Strong nuclear immunoreactivity is observed in osteoblasts and chondroblasts of the growth plate and in the cambium layer (box and corresponding inset). (m–n) Mouse embryo. Strong nuclear immunoreactivity is observed in cells with osteoblast and chondroblast morphologies. (n) Higher magnification of the boxed region in (m).

Canine, feline, and murine mature osteocytes and, to a lesser extent, quiescent chondrocytes/chondroblasts rarely demonstrated convincing Osx immunoreactivity (Figs. 2f and 3a, b). No labeling of canine osteoclasts (Fig. 3a) and parrotlet, chicken (Fig. 3h), or zebrafish (Fig. 3i) osteoblasts, osteocytes, and chondrocytes/chondroblasts was observed. In immature feline and mouse tissues, cells with osteoblast or chondroblast morphologies exhibited Osx immunoreactivity (Fig. 3l–n).

## Discussion

Definitive diagnosis of OSA in domestic animals can be highly challenging, especially in the event of very small amounts of and/or heterogeneously distributed osteoid. This is reflected in a number of recent studies that have attempted to characterize reliable markers, particularly those targeting malignant neoplastic osteoblasts in dogs.^1,4,35,42^ Whilst several markers, including alkaline phosphatase, osteocalcin, osteonectin, osteopontin, and RUNX2, have demonstrated very good sensitivity in identifying canine (or human) OSA, these markers typically have lower specificities in dogs.^4,11,35,42^ As a result, Barger et al suggested the application of antialkaline phosphatase and anti-RUNX2 antibodies in series as a sensitive (87%) and specific (85%) approach to the diagnosis of canine OSA. When used as part of an IHC panel, the anti-special AT-rich sequence-binding protein 2 antibody has a sensitivity of 86.0% and a specificity of 98.8% for OSA in dogs.^33^ In addition, Amaral et al^1^ suggested that a panel of antibodies against vimentin, osteonectin, and osteocalcin may represent a useful diagnostic and prognostic aid for canine OSA, although they did not indicate the sensitivity or specificity of this approach. When viewed within the context of these previous investigations, the present study demonstrates that IHC for Osx, applied as a single antibody, is a highly sensitive (92.5%) and specific (95.0%) marker for canine OSAs. Furthermore, combining this IHC marker with an established scoring and classification system was predictive and associated with good interobserver agreement (91.3% classification agreement). Therefore, the approach outlined in this study is proposed as a useful adjunctive tool in the diagnosis of canine OSA.

Reliable markers for feline OSAs have not been reported. In this study, IHC for Osx was also applied to feline samples and demonstrated an overall sensitivity of 70.0%, a specificity of 75.0%, and a correctly classified value of 72.5%, which was lower than for canine samples. Different scoring thresholds may improve the utility of Osx IHC for feline bone tumors; however, this is outside of the scope of this study and would likely require a larger study population.

Beyond the incidence, the age of onset, predilection sites, metastatic rates, and prognostic characteristics of OSAs vary between cats and dogs.^9^ These variations may suggest differences in the pathobiology of OSA between these 2 species, which could potentially include dissimilarities in binding epitopes and/or transcription factor expression and could therefore underlie the reduced efficacy of Osx IHC in cats compared to dogs. Further investigation of this hypothesis would require molecular characterization of feline Osx, which again lies outside the scope of this study.

The difference in sensitivity of Osx IHC in identifying canine and feline OSAs may also have been influenced by the relative proportions of each subtype within each OSA population, notwithstanding inherent challenges in subtyping OSAs. OSA subtypes were not equally represented across both species; for example, giant cell-rich OSA accounted for 25% of the total feline OSAs but was not included in the canine OSA study population. In dogs, productive osteoblastic OSAs are reportedly the most common OSA subtype. In one study, these accounted for 55% of the OSAs examined (67.5% of the OSAs in the current study), whilst poorly productive osteoblastic OSAs accounted for 14% (8% in the current study), chondroblastic OSAs accounted for 13% (7% in the current study), and giant cell-rich OSAs accounted for 2% (0% in the current study).^24^ Reports in cats vary. In 1 study, osteoblastic tumors comprised 42% of feline OSA; mixed osteoblastic and fibroblastic, chondroblastic, and/or telangiectatic subtypes combined accounted for a further 50%; and exclusively fibroblastic OSAs made up the remaining 8%.^9^ In a different study investigating 10 feline OSAs, 40% were chondroblastic, 30% were fibroblastic, and 30% were osteoblastic subtypes.^7^ The feline OSA study population in this study comprised roughly comparable proportions of productive (35%) and poorly productive (15%) osteoblastic OSAs, but fewer (15%) chondroblastic OSAs. Therefore, and whilst the exact proportions vary between studies, osteoblastic, in particular productive, OSAs, which would be expected to be more likely to express Osx, seem to be most common in both species, and the variation in incidence of productive osteoblastic subtypes between canine (67.5%) and feline (35%) cases in this study could have contributed to differences in the sensitivity of Osx immunoreactivity between dogs and cats.

In dogs, poorly productive osteoblastic OSAs were the most common subtype classified as non-OSA following IHC for Osx. In cats, productive, giant cell-rich, and telangiectatic subtypes were most commonly classified as non-OSAs following IHC for Osx; feline productive osteoblastic OSAs also had the greatest variability in scores. This most likely reflects the degree of divergence of the neoplastic population from functional osteoblasts, as suggested both by their limited production of osteoid and histological appearance. The neoplastic population in OSA is notoriously diverse, and therefore, lack of or aberrant protein expression is not unexpected.^11,12,16,29,42^

Productive osteoblastic OSAs in both cats and dogs typically demonstrated the strongest Osx immunoreactivity, likely reflecting a closer relationship of these neoplastic cells to functional osteoblasts, which is also suggested by their histological appearance and consistent production of osteoid. At the same time, both canine and feline chondroblastic OSAs frequently received high IHC scores, and chondrosarcomas were the CTRLs most commonly classified (score ≥ 2) as OSA in this study in both cats and dogs. Indeed, 4/5 feline chondrosarcomas were classified as OSAs following IHC for Osx. The most likely explanation for this is that chondrocytes and osteoblasts arise from a common bipotential progenitor known as a preosteoblast,^30^ and although Osx is thought to be critical for the differentiation of preosteoblasts into functional osteoblasts, *Osx* transcripts have been identified transiently in differentiating chondrocytes in murine embryos.^10^ Furthermore, Osx expression detected by IHC has been reported in chondrocytes in the mandibular cartilage of newborn mice.^48^ Therefore, the Osx immunoreactivity of chondrosarcomas in this study may be the result of recapitulation of (transient) Osx expression by neoplastic cells. An alternative explanation is that these tumors could indeed be chondroblastic OSAs that were misdiagnosed due to sampling bias and/or sectioning artifact, which precluded identification of osteoid. While this scenario cannot categorically be ruled out, the 2 canine chondrosarcomas classified as OSAs were both nasal, and a misdiagnosis of nasal chondroblastic osteosarcoma as nasal chondrosarcomas seems less likely given their relative frequencies.^25,31,44,46^

In comparison to the dogs, the location of 4/5 feline chondrosarcomas classified as OSA following IHC for Osx was more heterogeneous and included 1 nasal mass and 3 masses from the trunk and limbs, of which 1 was subcutaneous and the exact locations of the other 2 were unclear. Similarly, the 1 feline fibrosarcoma classified as an OSA following IHC for Osx originated from the truncal subcutis. Whilst again misdiagnosis cannot categorically be ruled out, especially as fibroblastic osteosarcomas can be challenging to differentiate from fibrosarcomas, the possibility of the immunoreactivity of the non-osteoblastic mesenchymal cells reflecting a common precursor is plausible, especially as osteoblastic and chondroblastic injection site sarcomas have been reported.^1,15,17,19,29^ On a further potentially associated note, 5/20 feline OSAs (25%) were present in the region of the scapula, of which 3 were associated with the bone and 2 were present in the overlying subcutis; no canine periscapular masses, including subcutaneous extraskeletal OSAs, were identified. Therefore, the possibility that these feline tumors could be vaccine-associated may need to be considered.

The neoplastic population in canine osteoma labeled positively for Osx, which is to be expected given the role of Osx in the differentiation of osteoblasts. The neoplastic population in canine multilobular tumor of bone is less well characterized; however, these tumors are thought to arise from precursors within the periosteum. This tumor also demonstrated some Osx immunoreactivity, which is expected given the production of cartilage and bone in such tumors.^24,40^

Osx expression was not only observed in cells with osteoblastic but also in cells with chondroblastic morphology in embryonic mouse and immature kitten bone, which is consistent with a role of Osx in bone development. Non-neoplastic mature canine, feline, and murine (mouse and rat) osteoblasts also demonstrated Osx immunoreactivity, as did osteoblasts in canine and feline reactive and woven bone formation. Therefore, IHC for Osx does not distinguish between normal, reactive, and neoplastic osteoblasts and would be expected to label reactive osteoblasts in the context of callus formation, considering its role in the physiological maturation of osteoblasts and homeostasis of adult bone.^3,30^ Importantly, Osx immunoreactivity would also be expected in metaplastic bone and possibly also cartilage formation, which must be considered when applying anti-Osx antibody (and this scoring system) to samples with such changes, such as malignant melanomas with intralesional metaplastic bone and/or cartilage formation. In accordance with the role of Osx in the differentiation of osteoblasts, osteocytes and quiescent chondrocytes/chondroblasts were considerably less immunoreactive to anti-Osx antibodies than osteoblasts.^30^ Osteoclasts lacked immunoreactivity.

The lack of Osx immunoreactivity observed in osteoblasts (and osteocytes) of birds (parrotlet and chicken) and a zebra-fish in this study may reflect their evolutionary divergence from mammals. While Osx is evolutionarily conserved across most vertebrates, its role and amino acid sequence seem to differ between species.^13,30^ *Osx*-null mice demonstrate embryonic failure of bone formation.^30^ The same is true in some fish, e.g. Medaka (*Oryzias latipes*).^47^ However, *Osx*-mutant carp (*Cyprinus carpio)* and, in other studies, zebrafish (reports vary) are able to mature to juveniles or young adults before bone lesions become appreciable.^8,23,30,47,49^ The absence of immunoreactivity in a zebrafish of unknown age, but presumed to be subadult or adult, in this study is difficult to interpret in the context of conflicting literature; however, a possible reduced reliance on Osx for early bone development in fish may need to be considered. Similarly, the importance of Osx in birds is uncertain; Yu et al^47^ evaluated 78 avian genomes, found no *Osx* homologue, and identified an *Osx*-like gene in just 7 avian species for which there was no clear phylogenetic basis. This peculiarity of the avian genome may explain the absence of labeling of the avian tissues assessed in this study.

Prolonged decalcification (>8 days) is reported to reduce nuclear immunoreactivity in some cases.^26^ We compared Osx immunoreactivity between paired formic acid decalcified and non-decalcified sections in a small subset of OSAs (*n* = 4). The non-decalcified tissues were given identical or slightly higher scores than decalcified tissues, but this did not change the classification (OSA vs non-OSA) in any case. Furthermore, should decalcification significantly impede immunoreactivity, lesions with a greater proportion of mineralized bone, such as productive OSAs, would be expected to require longer decalcification and therefore demonstrate reduced Osx immunoreactivity, whereas in this study, canine productive OSA labeled more strongly than less productive subtypes, although this may also be the consequence of dedifferentiated neoplastic cells as discussed above. Therefore, based on this study, IHC for Osx appears to be suitable for use on tissues decalcified with formic acid. Variable effects of different methods of decalcification on IHC have been reported; in particular, HCl decalcification is reported to decrease immunoreactivity of melanomas to anti-melanA and anti-PNL2 antibodies, and HCl-decalcified canine OSAs demonstrated weaker osteocalcin and osteonectin immunoreactivity compared to tissues decalcified with formic acid or EDTA.^32,42^ Therefore, the efficacy of anti-Osx antibody in the context of non-formic acid-based decalcification remains unclear, and the decalcification method ought to be considered when interpreting Osx immunoreactivity.

Similarly, bleaching did not affect the scoring of Osx expression in canine melanoma in this study. However, considering that melanomas would not be expected to label with anti-Osx antibody and that no bleached OSAs were evaluated, the possibility that bleaching OSAs may reduce Osx immunoreactivity cannot be excluded. Bleaching is reported to reduce immunoreactivity of some IHC markers. For example, melanA and HMB-45 immunoreactivity in canine melanoma was abolished after bleaching, but PNL2 was not.^32^ Regardless, this is unlikely to be of major concern, as predominantly only very poorly pigmented melanomas would be likely to be subject to IHC for Osx, and these are unlikely to require bleaching.

The retrospective nature of this study meant that there were several variables associated with sample handling that could have impacted the results of this study. Firstly, the age of the blocks; although no obvious association between block age and immunoreactivity score was observed. Secondly, the duration of fixation of these samples was unknown, and prolonged fixation is known to reduce immunoreactivity in some cases.^32^ This potential confounding factor is partially mitigated by the fact that the vast majority of study samples were biopsies submitted to one diagnostic facility; therefore, reasonable standardization of fixation and processing could be expected. Osteosarcomas sampled at necropsy (4/60) rather than biopsy would likely have more variable fixation times and prolonged sampling intervals; however, they did not exhibit appreciably decreased intensity of immunoreactivity. Finally, the time spent exposed to decalcification agents also varied between samples, reaching up to 14 days; however, as previously discussed, decalcification did not appear to affect Osx immunoreactivity in this study.

As discussed above, some subtypes of OSA were more common than others, and the number of samples and the distribution of subtypes differed between cats and dogs, which may compromise direct comparison between Osx expression in canine and feline OSAs. Overall, however, the number of samples in this study is greater than those reported in other studies examining IHC markers for OSAs. Considering that certain subtypes of OSA tended to exhibit a lower expression of Osx, it is advised that additional IHC tests, such as for endothelial markers in suspected telangiectatic OSAs, are performed alongside IHC for Osx where necessary.

The inherent difficulty in diagnosing OSA on hematoxylin and eosin-stained slides was the initial impetus for this investigation and is a significant potential limitation of the study. This was mitigated as far as possible by choosing study OSAs based on the definitive presence of osteoid formed by a malignant neoplastic population and further validating this selection via consensus diagnosis between 2, or if necessary 3, board-certified veterinary anatomic pathologists. Equivalent stringency was applied for the selection of non-OSA samples and other tissues examined. Nevertheless, the possibility that some of the CTRLs classified as OSA following IHC for Osx truly represented OSAs in which the production of osteoid by a malignant neoplastic population had not been detected due to sampling bias (including the submission of incisional biopsies) or sectioning artifact cannot categorically be ruled out.

Finally, the potential for recognition of study samples by the scorer involved in initial sample evaluation was mitigated by the blinded assessment of samples and concurrent assessment by a second scorer who had not been involved in initial sample evaluation. These 2 scorers assigned comparative scores (interscorer classification agreement 93.3%). There was minimal overlap (2 of 7 cases) between samples, both re-evaluated on hematoxylin and eosin and scored by the third reviewer/scorer. In general, standardized and unbiased scoring was achieved by annotation of the regions of interest by an individual not involved in scoring and statistical examinations conducted by an individual who was completely independent from sample evaluation.

In conclusion, the results of this study suggest that IHC for Osx, in combination with the scoring system described herein, is a highly sensitive and specific marker for canine OSA and a moderately sensitive and specific marker for feline OSA that can be used on samples decalcified with formic acid, warranting further, even larger-scale studies assessing this marker. Some OSA subtypes are less reliably immunoreactive than others, and particular care is advised when interpreting Osx immunoreactivity of suspected poorly productive canine and feline OSAs, as well as productive, giant cell-rich, and telangiectatic feline OSAs; supplemental relevant IHC antibodies could also be considered. On a similar note, immunolabeling of neoplastic chondrocytes with anti-Osx antibody was also observed; therefore, careful assessment for the presence of osteoid produced by a malignant neoplastic population, combined with taking into account the clinical features, remains key for discrimination between chondroblastic OSA and chondrosarcoma. In addition, since the anti-Osx antibody immunolabels all (canine, feline, and murine) osteoblasts, to a lesser extent osteocytes, and in some cases also chondroblasts and chondrocytes, care must be taken when evaluating Osx immunoreactivity in the context of callus formation, reactive bone formation, and osseous and chondroid metaplasia. Nonetheless, IHC for Osx is anticipated to be a useful tool to support the diagnosis of canine and feline OSA.

## Supplemental Material

sj-pdf-1-vet-10.1177_03009858261457962 – Supplemental material for Immunohistochemical detection of the transcription factor osterix in canine and feline osteosarcoma Supplemental material, sj-pdf-1-vet-10.1177_03009858261457962 for Immunohistochemical detection of the transcription factor osterix in canine and feline osteosarcoma by Katherine Ings, Stephanie Plog, Mark Jackson, Francesco Marchesi and Angie Rupp in Veterinary Pathology
