# Supplementary material for: Immunohistochemical detection of the transcription factor osterix in canine and feline osteosarcoma
Source: Vet Pathol. 2026 Jun 30;63(6):1013–24. doi: 10.1177/03009858261457962 (PMC13714195; doi:10.1177/03009858261457962)
Supplement: Supplementary file 1 [file sj-pdf-1-vet-10.1177_03009858261457962.pdf]

Supplemental Materials

Immunohistochemical detection of the transcription factor osterix in canine and feline osteosarcoma

Katherine Ings, Stephanie Plog, Mark Jackson, Francesco Marchesi, and Angie Rupp

Supplemental Table S1: Study data

Sample	Year	Type	Subtype	Scorer 1 (scan)	Scorer 2 (scan)	Scorer 2 (glass)	Scorer 3 (scan)	Location	IHC for diagnosis
D1	2022	CTRL	fibrosarcoma	0	0	0		bridge of nose (oral?)	n
D2	2021	CTRL	hemangiosarcoma	0	0	0		spleen	n
D3	2021	CTRL	fibrosarcoma	0	0	1		subcutis hip	n
D4	2021	CTRL	fibrosarcoma	0	0	0		orbit	n
D5	2021	OSA	productive	3	2	2		distal radius, R	n
D6	2021	CTRL	malignant melanoma	NE	0	0		ear tip	n
D6 BL	2021	CTRL	malignant melanoma	1	0	0		ear tip	n
D7	2021	CTRL	hemangiosarcoma	0	0	0		subcutis (perineal)	y
D8	2021	CTRL	hemangiosarcoma	0	0	0		spleen	n
D9	2021	CTRL	hemangiosarcoma	0	0	0		spleen	y
D10	2021	CTRL	chondrosarcoma	2	1	2	1	nasal	n
D11	2021	CTRL	malignant melanoma	NE	0	0		lip	n
D11 BL	2021	CTRL	malignant melanoma	1	0	0		lip	n
D12	2021	CTRL	malignant melanoma	NE	0	0		third eyelid	n
D12 BL	2021	CTRL	malignant melanoma	0	0	0		third eyelid	n
D13	2021	CTRL	malignant melanoma	NE	0	0		hindlimb, R	n
D13 BL	2021	CTRL	malignant melanoma	0	0	0		hindlimb, R	n
D14	2021	CTRL	chondrosarcoma	3	1	2	1	nasal	n
D15	2021	CTRL	fibrosarcoma	0	0	0		lip	n
D16	2020	OSA	chondroblastic	2	2	2		ileum	n
D17	2020	CTRL	fibrosarcoma	0	0	0		oral mucosa	n
D18	2020	OSA	fibroblastic	3	2	2		tibia, L	n
D19	2020	OSA	poorly productive	0	1	0		distal femur, R	n
D20	2020	CTRL	histiocytic sarcoma	0	0	0		subcutis near tarsus	y
D21	2020	OSA	productive	4	4	4		distal humerus, L	n

D21 ND	2020	OSA	productive	4	4	4		distal humerus, L	n
D22	2020	OSA	productive	3	2	2		distal femur	n
D22 ND	2020	OSA	productive	4	3	4		distal femur	n
D23	2020	OSA	chondroblastic	4	3	3		subcutis inguinal region	n
D24	2020	CTRL	malignant melanoma	NE	NE	1		oral mucosa	n
D24 BL	2020	CTRL	malignant melanoma	2	1	1	1	oral mucosa	n
D25	2019	CTRL	fibrosarcoma	1	0	1		subcutis face	n
D26	2019	CTRL	histiocytic sarcoma	0	0	0		subcutis shoulder	y
D27	2019	CTRL	hemangiosarcoma	1	1	1		muscle	n
D28	2019	OSA	productive	0	0	0		jejunum	y
D29	2019	CTRL	hemangiosarcoma	0	0	0		subcutis thorax	n
D30	2019	OSA	productive	4	3	3		distal radius, R	n
D31	2019	OSA	productive	4	2	3		forelimb, L	n
D32	2019	OSA	productive	3	2	3		humerus, R	n
D32 ND	2019	OSA	productive	4	3	4		humerus, R	n
D33	2019	CTRL	histiocytic sarcoma	0	0	0		eye	y
D34	2019	CTRL	hemangiosarcoma	1	1	1		muscle	y
D35	2019	CTRL	malignant melanoma	NE	0	NE		oral mucosa	n
D35 BL	2019	CTRL	malignant melanoma	0	0	1		oral mucosa	n
D36	2019	OSA	productive	2	1	1	2	proximal humerus, R	n
D37	2019	OSA	productive	4	3	3		proximal humerus, L	n
D38	2019	OSA	productive	3	2	2		proximal humerus, L	n
D39	2019	CTRL	fibrosarcoma	0	0	0		oral mucosa	y
D40	2019	CTRL	hemangiosarcoma	0	0	0		spleen	n
D41	2018	CTRL	histiocytic sarcoma	0	0	0		joint	y
D42	2018	OSA	productive	4	3	3		distal femur, L	n
D43	2018	CTRL	chondrosarcoma	2	2	2		nasal	n
D44	2018	CTRL	histiocytic sarcoma	0	0	0		thigh, R	y
D45	2018	OSA	chondroblastic	4	2	2		proximal humerus, L	n
D46	2018	OSA	productive	4	3	3		tibia, R	n

D47	2018	OSA	chondroblastic	4	4	3		spleen	n
D48	2018	OSA	productive	3	2	2		spleen	n
D49	2018	OSA	productive	3	1	1	2	proximal humerus, L	n
D50	2018	CTRL	fibrosarcoma	0	0	0		mandible	n
D51	2018	OSA	telangiectatic	3	2	2		proximal humerus, L	n
D52	2018	OSA	productive	4	3	2		distal femur, L	n
D53	2017	OSA	productive	3	3	3		distal radius, R	n
D54	2017	OSA	chondroblastic	3	4	4		distal ulna, R	n
D55	2017	CTRL	chondrosarcoma	0	0	0		nasal	n
D56	2017	OSA	productive	4	3	4		chest	n
D57	2017	CTRL	chondrosarcoma	1	1	1		nasal	n
D58	2017	OSA	productive	4	2	3		oral	n
D59	2017	OSA	productive	2	2	2		zygomatic arch	n
D60	2017	CTRL	malignant melanoma	NE	0	0		oral mucosa	n
D60 BL	2017	CTRL	malignant melanoma	0	0	0		oral mucosa	n
D61	2017	OSA	fibroblastic	2	1	1	2	proximal humerus, R	n
D62	2017	OSA	productive	3	2	2		oral	n
D63 N	2016	OSA	productive	3	2	2		spine	n
D64	2016	OSA	productive	3	3	2		distal radius	n
D65 N	2016	OSA	productive	4	3	2		vertebral	n
D66	2016	OSA	fibroblastic	3	2	1		metatarsus	n
D67	2016	CTRL	histiocytic sarcoma	0	0	0		lung	y
D68 N	2016	OSA	chondroblastic	3	2	2		skull	n
D69	2016	CTRL	histiocytic sarcoma	0	0	0		brain	y
D70	2016	CTRL	malignant melanoma	NE	0	0		eye	n
D70 BL	2016	CTRL	malignant melanoma	0	0	0		eye	n
D71	2016	OSA	productive	3	2	2		distal radius, R	n
D72	2015	OSA	poorly productive	4	4	3		mandible, R	n
D73	2015	CTRL	chondrosarcoma	1	1	2		nasal	n
D74	2015	CTRL	chondrosarcoma	0	0	0		nasal	n

D75	2015	OSA	productive	4	3	3		femur, R	n
D76	2015	OSA	productive	3	1	2	2	distal tibia	n
D77	2014	CTRL	histiocytic sarcoma	0	0	0		spleen	y
D78	2014	CTRL	chondrosarcoma	3	3	3		nasal	n
D79	2014	OSA	poorly productive	1	1	0		distal femur, L	n
D80	2013	OSA	productive	2	2	1		maxilla	n
C1	2024	CTRL	histiocytic sarcoma	0	0	0		liver	y
C2 N	2021	CTRL	histiocytic sarcoma	0	0	0		spleen	y
C3	2021	CTRL	chondrosarcoma	3	3	3		nasal	n
C4	2021	CTRL	fibrosarcoma	1	1	1		subcutis injection site	n
C5	2021	CTRL	fibrosarcoma	1	1	1		dorsal neck (likely subcutis)	n
C6	2020	CTRL	hemangiosarcoma	0	0	0		subcutis of abdominal wall	n
C7	2020	OSA	productive	3	3	2		proximal tibia, R	n
C8	2020	OSA	productive	4	4	4		subcutis over scapula	n
C9	2020	CTRL	fibrosarcoma	2	2	1		subcutis lateral flank, L	n
C10	2019	OSA	poorly productive	3	2	2		distal scapula, R	n
C11	2018	CTRL	fibrosarcoma	1	0	1		periarticular (tarsus)	n
C12	2018	OSA	chondroblastic	4	3	3		hindlimb	n
C13	2018	OSA	productive	0	1	0		chest	n
C14 N	2018	OSA	giant cell	1	0	1		skull	n
C15	2018	OSA	poorly productive	2	2	2		skull	n
C16	2018	OSA	giant cell	2	2	1		pelvis, R	n
C17	2017	CTRL	fibrosarcoma	1	1	1		subcutis between shoulders	n
C18	2017	OSA	giant cell	1	1	1		distal femur, L	n
C19	2016	OSA	productive	3	3	3		scapula	n
C19 ND	2016	OSA	productive	4	3	4		scapula	n
C20	2016	CTRL	hemangiosarcoma	0	0	0		subcutis (axilla)	n
C21	2015	CTRL	hemangiosarcoma	0	0	1		subcutis	n
C22	2014	CTRL	hemangiosarcoma	1	0	0		subcutis	n

C23	2014	CTRL	chondrosarcoma	3	2	3		subcutis flank	n
C24	2013	CTRL	malignant melanoma	NE	NE	NE		skin	n
C24 BL	2013	CTRL	malignant melanoma	0	0	0		skin	n
C25	2013	CTRL	chondrosarcoma	2	2	2		hindlimb	n
C26	2013	CTRL	chondrosarcoma	0	0	0		metacarpus, R	n
C27	2013	CTRL	hemangiosarcoma	0	0	0		spleen	n
C28	2012	OSA	telangiectatic	0	0	0		forelimb	n
C29	2012	OSA	telangiectatic	1	1	1		proximal humerus, R	n
C30	2010	CTRL	malignant melanoma	NE	0	0		skin on foot	n
C30 BL	2010	CTRL	malignant melanoma	0	0	0		skin on foot	n
C31	2010	OSA	chondroblastic	3	3	3		scapula	n
C32	2010	CTRL	chondrosarcoma	2	3	3		axilla	n
C33	2010	OSA	chondroblastic	3	3	4		Interscapular, likely subcutis	n
C34	2009	OSA	productive	0	0	0		oral	n
C35	2009	OSA	giant cell	3	3	3		carpus, R	n
C36	2008	OSA	productive	4	3	2		distal femur, L	n
C37	2008	CTRL	malignant melanoma	NE	0	0		eye	n
C37 BL	2008	CTRL	malignant melanoma	0	0	0		eye	n
C38	2008	OSA	giant cell	4	3	4		skull	n
C39	2006	OSA	poorly productive	2	2	2		distal tibia	n
C40	1999	OSA	productive	3	2	2		head	n

BL– bleached; C – cat; CTRL – control; D – dog; IHC – immunohistochemistry; N – necropsy sample; NE – not examined; ND – non-decalcified; OSA – osteosarcoma. (Please note C14 (necropsy sample) and C15 (biopsy sample) represent samples taken from the same animal yet at different timepoints.)
